# Applying the Implementation Research Logic Model (IRLM) as a research planning tool in a multi-project, rural health initiative to improve cardiovascular health service delivery outcomes and quality

**DOI:** 10.21203/rs.3.rs-10584584/v1

**Published:** 2026-10-06

**Authors:** Sarah Masyuko, Sarah Gimbel, Brian Chan, Todd Korthuis, Audrey L. Blewer, Hayden B. Bosworth, Monique Starks, Angela G. Spencer, Sarah de Loizaga, Andrea Beaton, Anju Sahay, Paul Heidenreich, Katrina F. Ortblad, Justin D. Smith, Chris T. Longenecker

**Affiliations:** 1 Department of Global Health, University of Washington, Seattle, Washington, United States of America; 2 Department of Child, Family and Population Health Nursing, School of Nursing, University of Washington, Seattle, Washington, United States of America; 3 Department of Medicine, Section of Addiction Medicine, Oregon Health & Science University, Portland, Oregon, United States of America; 4 Department of Family Medicine and Community Health Duke University School of Medicine, Durham, North Carolina, United States of America; 5 Department of Population Health Sciences Duke University School of Medicine, Durham, North Carolina, United States of America; 6 Duke University School of Medicine, Durham, North Carolina, United States of America; 7 Heart Institute, Cincinnati Children’s Hospital Medical Center, Cincinnati, Ohio, United States of America; 8 University of Cincinnati, Department of Pediatrics, Cincinnati, Ohio, United States of America; 9 United States Department of Veterans Affairs, Veterans Affairs Palo Alto Health Care System,Palo Alto, California, United States of America; 10 Department of Medicine, Stanford University, Stanford, California, United States of America; 11 Public Health Science Division, Fred Hutchinson Cancer Center, Seattle, USA; 12 Department of Population Health Sciences, Division of Health System Innovation and Research, Spencer Fox Eccles School of Medicine at the University of Utah, Salt Lake City, Utah, USA; 13 Division of Cardiology, University of Washington, Seattle, Washington, United States of America

**Keywords:** Implementation Research Logic Model, Rural PRO-CARE, Health Equity Research Network, Rural, Cardiology, Implementation science, Reciprocal Innovation, United States

## Abstract

**Background:**

Increasingly, domestic and global initiatives are being designed to build synergistic networks of leading researchers to tackle major health challenges. In this case study, we applied the Implementation Research Logic Model (IRLM) to individual projects to enhance collaboration, learning and knowledge generation across a large research network.

**Methods:**

The ReciPROcal Innovations to Improve Cardiovascular Care in Rural America (Rural PRO-CARE) network, is a five-project initiative funded by the American Heart Association. The network used the IRLM to facilitate implementation planning and learning across projects testing a variety of implementation strategies to improve cardiovascular health outcomes in rural America. A collaborative case study approach identified determinants, implementation strategies, mechanisms and outcomes for each project using the Consolidated Framework for Implementation Research (CFIR), the Expert Recommendations for Implementing Change (ERIC), and Implementation Outcomes Framework (IOF) respectively. Projects developed causal pathway diagrams to define mechanisms of action for their specified implementation strategies. Semi-structured group interviews using an After-Action Review (AAR) approach, elucidated each teams’ understanding of how the IRLM worked, what was successful and how the process could improve.

**Results:**

Within the first year of the initiative, each project developed an IRLM which provided a visual and theoretical structure for planning, implementation and evaluation. This process identified similarities and differences across projects’ determinants, strategies and outcomes. The most frequently identified barriers to effective implementation of cardiovascular health strategies in rural America were high care and insurance costs, complex cardiovascular intervention workforce limitations, limited scope of practice, misaligned external policies, conflicting patient beliefs/knowledge, and lack of readiness for change. The most frequently identified implementation outcomes across projects were reach, followed by acceptability and sustainability/maintenance. From the AAR, all project teams noted greater ease and use of common implementation language and reported linking facilitators and barriers to strategies, mechanisms, and intended outcomes deepened team members’ alignment on project goals. New strategies to overcome barriers were highlighted through the IRLM process and specific measures were selected to assess implementation.

**Conclusion:**

The IRLM strengthened cross-site learning by making common determinants and synergies visible across projects enabling the network to target shared challenges more strategically and improve coordination across the initiative.

## Background

Increasingly, domestic and global initiatives are deliberately investing in the formation of research networks of leading researchers with the aim of advancing solutions to the most persistent and complex health challenges. This approach typically pools funding sources and then proposes a larger overarching problem or question that teams of scientists and/or practitioners propose studies to address these concerns. Oftentimes, the enthusiasm generated from these combined efforts is further buoyed by novel and synergistic collaborations across donors, including federal, bi-lateral or multi-lateral entities, foundations and industry sources. Examples of such initiatives include those supported by the U.S. National Institutes of Health, such as the NHLBI HLB-SIMPLe Consortium, the Disparities Elimination through Coordinated Interventions to Prevent and Control Heart and Lung Disease (DECIPHeR) Alliance, and the NCI Moonshot IMPACT Consortium^1–5^, as well as private foundations such as the American Heart Association’s (AHA) Health Equity Research Networks (HERN)^6^ and the Strategic Focus Research Networks (SFRN)^7^, among others.

A myriad of benefits can be generated by bringing together and fostering cross-site learning among diverse and qualified teams working to address common research questions beyond pooling resources.^8^ Notably, funded projects can: 1) learn from one another via dedicated forums throughout the funded research period; 2) discover insights across projects about what does and doesn’t work in different contexts; 3) design sustainable and scalable solutions in real-time, including data platforms and other forms of system interoperability informed by sharing of best practices; 4) identify and promote common tools to measure impact; and 5) identify future research questions and approaches.

To facilitate success across multi-site initiatives, research teams benefit from using shared methods and a common language to explain and describe their contexts, solutions and implementation realities. There is tremendous opportunity for research initiatives to cooperatively learn across studies by using common implementation science lexicon and frameworks. The Implementation Research Logic Model (IRLM) is one such tool.^9^ The IRLM encourages users to structure their studies into a logic model based on multiple frameworks and theories of implementation research that identifies implementation determinants and their relation to defined strategies, outcomes (implementation, service and clinical), and mechanisms connecting strategies to outcomes–underlying causal paths that constitute the theory or logic of change.

The AHA-funded Health Equity Research Networks (HERNs) were launched in 2021 as a funding mechanism to support cross-site research to improve access to equitable cardiovascular health care across the United States of America (US). The ReciPROcal Innovations to Improve Cardiovascular Care in Rural America (Rural PRO-CARE) network is a HERN, launched in July 2023.^10^ The Rural PRO-CARE network aims to develop contextually appropriate health innovations that increase access to evidence-based cardiovascular care amongst rural Americans. This case study documents the Rural PRO-CARE network’s use of the IRLM to facilitate implementation planning and catalyze learning across five projects, each implementing distinct implementation strategies to achieve the common aim of improved reach, acceptability, equity, uptake and sustainment of cardiovascular health care services in rural settings.

## Methods

A collaborative case study design was employed to describe the determinants, implementation strategies, and outcomes for each Rural PRO-CARE project. Project-specific results were shared throughout the yearlong process and descriptions of each project’s implementation approach and assumptions were developed using common terminology. The case study data was collected at two time points; 1) at group-based stakeholder workshops, where project-specific IRLMs were developed and shared across the projects, and 2) during endline group interviews, with project leads and implementation science experts, An After-Action Review (AAR) approach-a learning-focused collaborative assessment focused on improvement–was used in the group interviews to review the process, results, and priorities uncovered using by the IRLM.^11^

The IRLM combines and organizes the use of multiple frameworks to guide specification (e.g. clear description) of the determinants that inform the selection of implementation strategies and influence the mechanisms by which the intended implementation, service and clinical (patient-level) outcomes are achieved (Figure 1).^9^ In this multi-project initiative, the IRLM was used to enhance the transparency and reproducibility of findings across projects and allow the network members to compare heterogeneous projects in similar, comparative terms. This type of synthesis across projects is a specified means of using the IRLM^9^ that has been done in a case study implementing similar interventions and in systematic reviews of similar implementation projects.^12,13^

### Case Study Presentation

The Rural PRO-CARE network has the overarching goal of improving cardiovascular health equity and reducing cardiovascular disease in rural America. The five Rural PRO-CARE HERN projects are developing and testing different technology-engaged implementation strategies to promote uptake and sustainment of evidence-based interventions within local rural practice in the US over four years. They include: 1) GROW-Rural: A Global to Rural Innovation Network to Adapt Evidence Based Cardiovascular Interventions to Context; 2) PHARM-HF: Implementation and Evaluation of Pharmacist-Based Management of Chronic Heart Failure for Rural Veterans; 3) IN-STEP: Implementation and Scale-up of the American Indian Structural Heart Disease Partnership; 4) PEER-Heart: Rural Community Peer Partnerships for Improving Methamphetamine-Associated Heart Failure Screening and Engagement; and 5) RESTORe-CARE: Developing and Testing Drone-Delivered AEDs for Cardiac Arrests In America (Figure 2). These projects are led by researchers and practitioners from the University of Washington (UW), Palo Alto Veterans Affairs/Stanford University, Cincinnati Children’s Hospital, Duke University, and Oregon Health & Science University (OHSU).

Implementation of the Rural PRO-CARE projects is happening across geographically diverse areas of the US, with activities in the rural Alaska, Mountain West, West Coast, Southwest, and Southeast regions. The projects work across diverse rural health systems, including the Indian Health Service and Tribal Health Boards, the Department of Veterans Affairs, prehospital setting, and private clinics/hospitals. Delivery care points vary and include community health outreach sites, pre-hospital systems of care, pharmacies, emergency departments, and virtual via telemedicine and telehealth sites. Target project actors include pharmacists, community health workers, emergency medical responders, law enforcement, primary care providers, cardiologists, implementation scientists and fellows. Across this multidisciplinary network, diverse cardiovascular health challenges are addressed, with notable engagement of communities using a range of participatory research approaches. The Rural PRO-CARE network is specifically committed to reciprocal innovation to intentionally cross-pollinate and adapt effective solutions from one setting to improve health in another through idea co-development and exchange.

The UW hosts a Coordinating Center which supports the design, implementation, and dissemination of the work of this HERN research network. The Coordinating Center has three cores: 1) Administrative, 2) Training, and 3) Health Services and Implementation Research; the latter core supported the cross-site IRLM work.

### Cross-site IRLM implementation and evaluation

The process by which implementation science theories, models, frameworks and tools were applied and shared across Rural PRO-CARE projects using the IRLM, and the results of an evaluation by users via an AAR approach is presented below.^11,14^

#### 1. Module based workshops for IRLM development

Six module-based workshops were held from December 2023 to March 2024 to guide the network on how to develop and use the IRLM (Figure 3). Five modules introduced the IRLM and provided critical skill building on implementation science questions, frameworks, strategies, and outcomes. Each workshop comprised a short didactic session led by the implementation science core, followed by project teams presenting their draft IRLMs.

Within the use of the IRLM, we introduced and encouraged each project to use elements of the original Consolidated Framework for Implementation Research (CFIR)^15^, Expert Recommendations for Implementing Change (ERIC)^16^, and Implementation Outcomes Framework (IOF)^17^ as well as specification techniques to describe their implementation determinants, strategies, and outcomes.^18^ After each workshop module, project-specific team meetings were held, led by Coordinating Center implementation science leads, project lead investigators and implementation science-focused project team members, to complete and refine the IRLM. Implementation science support from the Coordinating Center was provided as needed.

The five workshop modules included

##### Module 1: The IRLM.

The IRLM was introduced. The organizational structure and intent of the tool were explained. Case examples were shared that provided ample opportunity for projects to relay questions related to deployment of the tool for their project. An explicit emphasis was placed on revisiting the IRLM regardless of whether the projects were in early, mid, or later implementation stages.

##### Module 2: Implementation outcome determinants.

Determinants to anticipated implementation outcomes were identified using existing literature and related preliminary data available to the project teams. We categorized key determinants under the five CFIR domains: Intervention Characteristics, Inner Setting, Outer Setting, Individuals Characteristics, and Process. Project teams were encouraged to identify determinants as barriers or facilitators with minus and plus signs, respectively.

##### Module 3: Implementation strategies.

Determinants were matched to implementation strategies for each project. Guided by the ERIC, strategies were carefully specified using the Proctor et al’s strategy to elucidate the actor (who delivers the strategy), action (what is the action done as part of the strategy), action target (which barriers/facilitators are targeted through the strategy), temporality (what order or sequence the strategy follows), and dose (what is the intensity of the strategy’s delivery–i.e., frequency and timing). ^18^

##### Module 4: Implementation outcomes.

Each project then outlined the targeted outcomes of the selected strategies. Proctor’s Implementation Outcomes Framework provided a standardized way to define project outcomes (implementation, service, or clinical). Special emphasis was devoted to reach and equity outcomes, which are central objectives for this rural cardiovascular health initiative. Each project shared the theories, models, and/or frameworks they planned to employ to design implementation plans allowing us to capture overlapping areas of harmonization and synergy across projects.

##### Module 5: Mechanisms.

Using the Causal Pathway Diagrams toolkit^19,20^ for selecting, tailoring, and optimizing implementation strategies, guided by the core, each project team mapped the causal logic (implementation strategy, mechanism, determinant, outcome) and moderators and preconditions (i.e., the elements that affect whether and how strategies work). Color coding was used across the IRLM to demonstrate how each team linked determinants to selected strategies, mechanisms, and outcomes.

This work culminated in each project presenting its IRLM at a joint in-person HERN meeting, where feedback was provided by network members and AHA technical advisors. In addition, we extracted the implementation determinants from each IRLM in a matrix to identify similarities and differences across projects.

#### 2. AAR Synthesis

After the semiannual HERN meeting presentations in April 2024, the coordinating committee gauged the perceptions of implementation science work within the Rural PRO-CARE projects and identified implementation science priorities for the second year of the four-year initiative. Five semi-structured group interviews were held with 2-5 study leads and implementation science fellows from each project. After establishing what was expected to happen, we explored with each team what actually occurred, why similarities/differences happened, and what could be improved in the future. A standard interview guide is presented in Table 1.

All interviews were conducted virtually via Zoom or Microsoft Teams, audio recorded, and transcribed. The transcripts were independently coded by two implementation scientists (SG and SM) and coding discrepancies were resolved through discussion and consensus. We used thematic analysis to identify and interpret themes within and across the data. Quotes and associated codes representing key themes were identified through consensus with the research team and presented to the network members for discussion and consensus.

## Results

### IRLM

After completion of the five orienting modules, each of the five HERN projects developed an IRLM. Although each project’s IRLM is distinct in terms of scope, content, context and approach, some similarities are present. For example, each IRLM included focus on expanding reach and improving equity across target populations.

The IRLM from the PEER-HEART (OHSU) project (Figure 4) illustrates specification of one implementation strategy and the accompanying causal pathway diagrams. Color-coding was used to highlight how determinants informed the selected implementation strategies, and in turn, how they influenced the mechanism and outcomes, providing visualization of a hypothesized pathway. IRLMs for the remaining four projects are annexed (Additional file 1).

#### Summary of implementation determinants across and outcomes.

Across the five Rural PRO-CARE projects, 102 non-unique determinants were identified within CFIR domains. The most frequently cited barriers to improving cardiovascular health in rural populations included high costs of care or insurance, the complexity of CVD interventions, workforce challenges (e.g., workload, training, scope of practice), external policy constraints, and patient-level factors such as beliefs, knowledge, and readiness for change. These are critical leverage points to develop implementation strategies to address these determinants at the network level.

The most reported implementation outcomes in the IRLM were: reach (5 projects), acceptability (4), sustainability/maintenance (4), feasibility (3), fidelity (3), cost (2), appropriateness (2), and adoption (2).

### AAR findings

The major thematic findings from the AAR are presented across the domains of what occurred, what went well, and where improvement was needed moving forward from 15 respondents in 5 group interviews across the network.

#### 1. What occurred?

Through collaborative, sequential skills-building workshops, the network’s teams applied common implementation science-informed language with the express purpose of sharing cross-site work. Respondents shared that it was helpful to identify drivers, strategies, outcomes, and proposed mechanisms of action both within their project and across sites.

“It’s, you know, hundreds of models and frameworks and theories, and it’s just really nice to have this, like planning tool to kind of like lay it all out and see how everything is sort of connected and to look at that and say, oh. this looks really good.” - Implementation science expert, PHARM-HF.

The IRLM process was viewed as good foundation for future implementation work. It is dynamic and iterative, allowing for further updates with new information.

“It feels hard to focus on how it’s being implemented when we’re having to learn…With development of the intervention and when we get to the point where we are ready to translate it to others, I think that’s where everything all the groundwork you all are laying becomes really important and maybe even with the second site.” - Principal investigator, RESTOReCARE

### What worked and what drove similarities/differences across sites?

IRLM work was deemed relevant and helpful in organizing each research team’s approach to developing their proposed implementation strategies and study protocols. It aided prioritization of key implementation strategies and outcomes and identified missing elements.

“- And to me, using that approach you know. Really [IRLM] brings clarity to the mind. It’s like you have kitchen tools but let’s organize them into drawers. And that’s what we always do. You know, we put the spoons here and we put a Saran wrap in another one and then everything. The knives go separately, so that’s what it is. The IRLM helps you to think and prioritize the variables. And what is missing? Which should be highlighted.” - Implementation science expert, PHARM-HF.

Ultimately the IRLM supported planning from project pre-implementation to sustainment and helped teams identify key stakeholders to engage in the process.

“So with that (IRLM) you can then get different pieces done. You know the pre implementation work, the implementation work brings in the right key stakeholders, so it helps everything” .” - Implementation science expert, PHARM-HF.

The IRLM process fostered conversations within and across the project teams including the need to expand the implementation strategies to address new determinants.

“- And so I think that the whole process really helped us think more specifically about the app so the m-health patient management application is only one piece of what we will probably ultimately test. Since we did the IRLM exercise we have had more conversations and more ideas about a provider facing component of our strategy which is something completely different. So, we’ll probably want to kind of go back and revisit the whole IRLM that we created and add maybe a new one…” - Principal investigator, GROW RURAL

This process was useful in detailing how the implementation strategies achieve desired outcomes. The specification of the implementation strategies was key to identifying missing actors and action targets.

“Well, I think it forces you to think more carefully more detailed about the strategy, first of all. And then we realized that our strategy was multiple components and the strategies are still being developed. Really, we haven’t done the hard work of clearly designing the various pieces of what that strategy will look like but that will be done over the next 6 months or so.” - Principal investigator, GROW RURAL

“I think that was helpful and thinking about, you know, standardization across projects. I think that we had ideas in place, but given the IRLM process and everything, I think that it allowed us to kind of facilitate thinking about the different aspects more I guess you could say in unity to what everybody else was doing, and I think it brought up some things that we hadn’t thought about previously.” - Implementation science expert, RESTORe CARE

“I think it was definitely useful and thinking about a tool that could be implemented, you know more broadly through the HERN and thinking about how each of us were collecting data and thinking about our processes and implementation strategies more. Unified as far as like the larger group is concerned.” - Implementation science expert, RESTORe CARE

The process of learning about and constructing an IRLM gave the project and entire RURAL PRO-CARE network a common language.

“The process of learning about and constructing an IRLM gave us a common language. Previously I had just thought of IS as only a way to develop and refine tools. But this IRLM creation process showed me how much more it can be. It took a broader theoretical approach.” -Principal investigator, PEER HEART

It also led to a deeper understanding of the projects.

“It helped us get on the same page. To sit down and look at all of our interventions and what we expected things, what we expected our implementation to look like, and how that might play out, or what we expected an outcome to be. And how did that tie into where it was in the beginning… and I began to understand our project better.” – Principal investigator, INSTEP

The IRLM improved cross-site learning while improving rigor, visibility and transparency across the projects.

“You know, it does help, really enhance kind of the rigor and some of the transparency and whatnot, across the projects and how we are doing things.” – Fellow, RESTORe CARE

Through the process, the IRLM helped to prioritize knowledge gaps and how to address them.

“Individually, we address them (knowledge gaps) there on the educational forum monthly meetings, first Fridays of the month. Tomorrow, we have a meeting so just to generate knowledge and know-how and experience in this field because this is management of heart failure patients, is new for the pharmacist.” – Implementation science Expert, PHARM-HF

### What can be improved next time?

The themes that emerged as to how the teams plan to use the IRLM moving forward are presented in Table 2.

## Discussion

This case study demonstrates that the IRLM can serve as a practical tool for aligning heterogeneous implementation projects within a research network, enabling shared language, cross-site comparison, and identification of common determinants and strategies.By jointly walking through the IRLM development process, the network developed project-specific baseline IRLMs that provided a summary visual representation of the teams’ implementation plan. It provided a shared lexicon to design and justify complex studies working toward improved cardiovascular disease care access in rural settings. Furthermore, guided by the CFIR, the IRLM provided a deep understanding of barriers and facilitators to achieving study outcomes across projects. This detail guided selection and specification of strategies to address those barriers and illustrated a testable causal pathway diagram for the interconnected strategies. In addition, it allowed network members to see similarities and differences in implementation determinants, strategies, and outcomes, facilitating learning between projects. This promoted cross-site sharing and learning around choice of outcome measures and strategies and provided visibility into other projects which was a catalyst of collaboration.

The IRLM has been used in consortiums with common interventions, which likely facilitates synthesis opportunities compared to more heterogeneous projects.^21^ Successful use of the IRLM in the Rural PRO-CARE network is reflected in its ability to provide a shared structure for planning, clarify implementation strategies and causal pathways, guide measurement, and facilitate cross-project learning and iterative refinement over time. It allowed a practical way to center implementation science in each project. For the Rural PRO-CARE network, it helped the project teams focus their work around the goal of improving delivery in rural cardiovascular health. It provides a baseline that becomes the reference for regular check-in points across each year. It is useful as a planning tool to aid appropriate strategy sequencing, outcome measure selection, and context tailoring. The IRLM serves as a dynamic blueprint, documenting each project’s implementation over the life of the award. Implementing the IRLM is feasible and typically requires modest training and facilitation upfront, a few focused team meetings, and iterative revision as implementation data emerge.

The IRLM increases visibility into other projects’ strategies and outcomes, promoting collaboration on study plans, design choices and outcome measurement. This process of network-wide IRLM development increased transparency and allowed projects to identify similarities and differences in implementation determinants, strategies, and outcomes, fostering shared understanding and increased project synergy. The outcome of this process has been actualized in a new round of studies with funding from the Rural PRO-CARE coordinating center where team members from different Rural PRO-CARE projects identified synergy between their projects, submitted a joint proposal known as a “synergy grant”, and work together to implement new projects to improve cardiovascular health care in the rural US. In addition, we identified common challenges working in cardiovascular health that the network is teaming up to address, such as clarification on scopes of practice of health providers in rural settings.

A strength of this case study is using the IRLM in a large research network, allowing application of the IRLM in projects with varied implementation strategies used in heterogeneous settings to achieve the common goal of improving cardiovascular health in rural populations. This allowed identification of overlapping thematic areas, synergies, or determinants across projects that the network could leverage or target support more effectively. While the IRLM is a useful planning tool, a limitation was that the IRLM creation process can be time and resource intensive, especially when coordinating with multiple teams with varied implementation science experience. It was also challenging to specify implementation strategies and present multiple proposed mechanisms for multiple strategies within one IRLM. One way this challenge was overcome was to include an addendum section to the IRLM that details the specification of each strategy and the accompanying causal pathway diagrams.

## Conclusions

The IRLM is a practical tool for creating a shared language and organizing implementation work across diverse projects in a research network. Its value is not only in clarifying strategies, determinants, mechanisms, and outcomes, but also in supporting iterative refinement as new implementation data and contextual changes emerge. In this way, the process of developing and revising the IRLM promotes coordination, shared learning, and more deliberate attention to how implementation is expected to lead to improved health outcomes.

## Supplementary Material

1

## Figures and Tables

**Figure 1:**
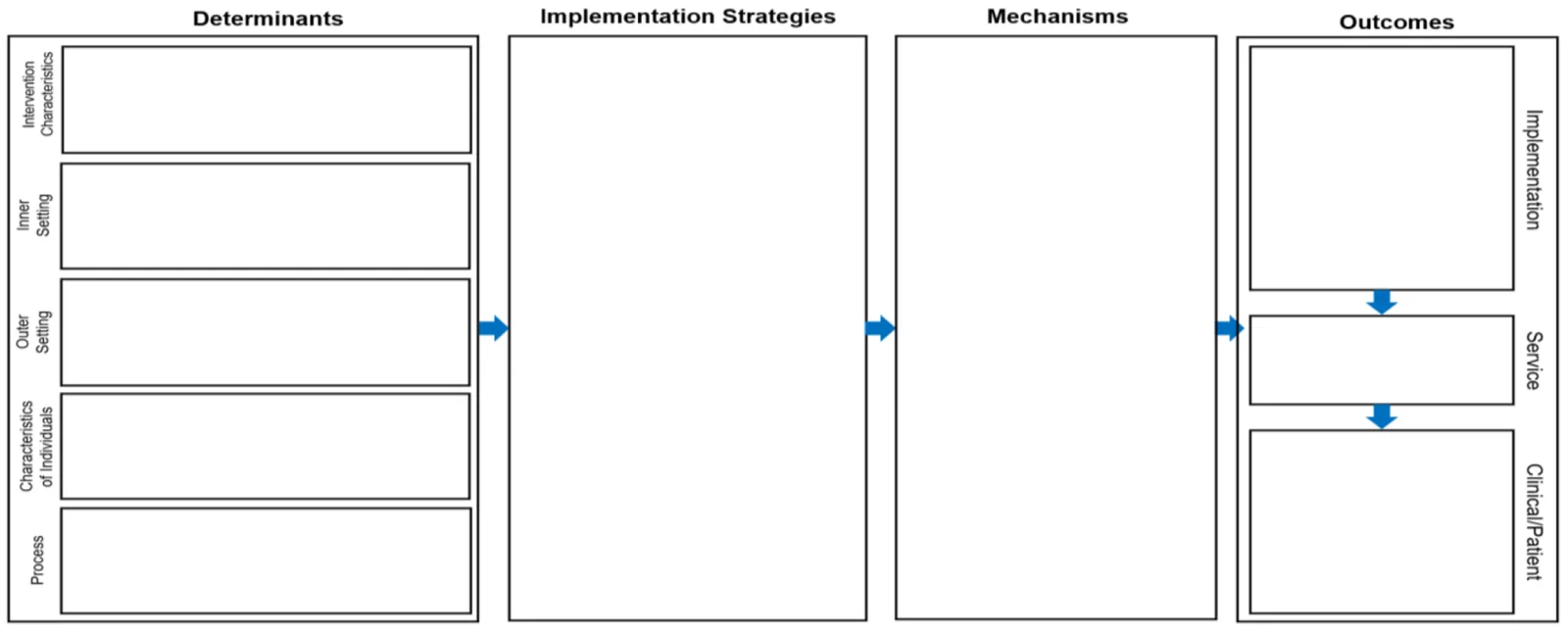
Implementation Research Logic Model template^9^

**Figure 2:**
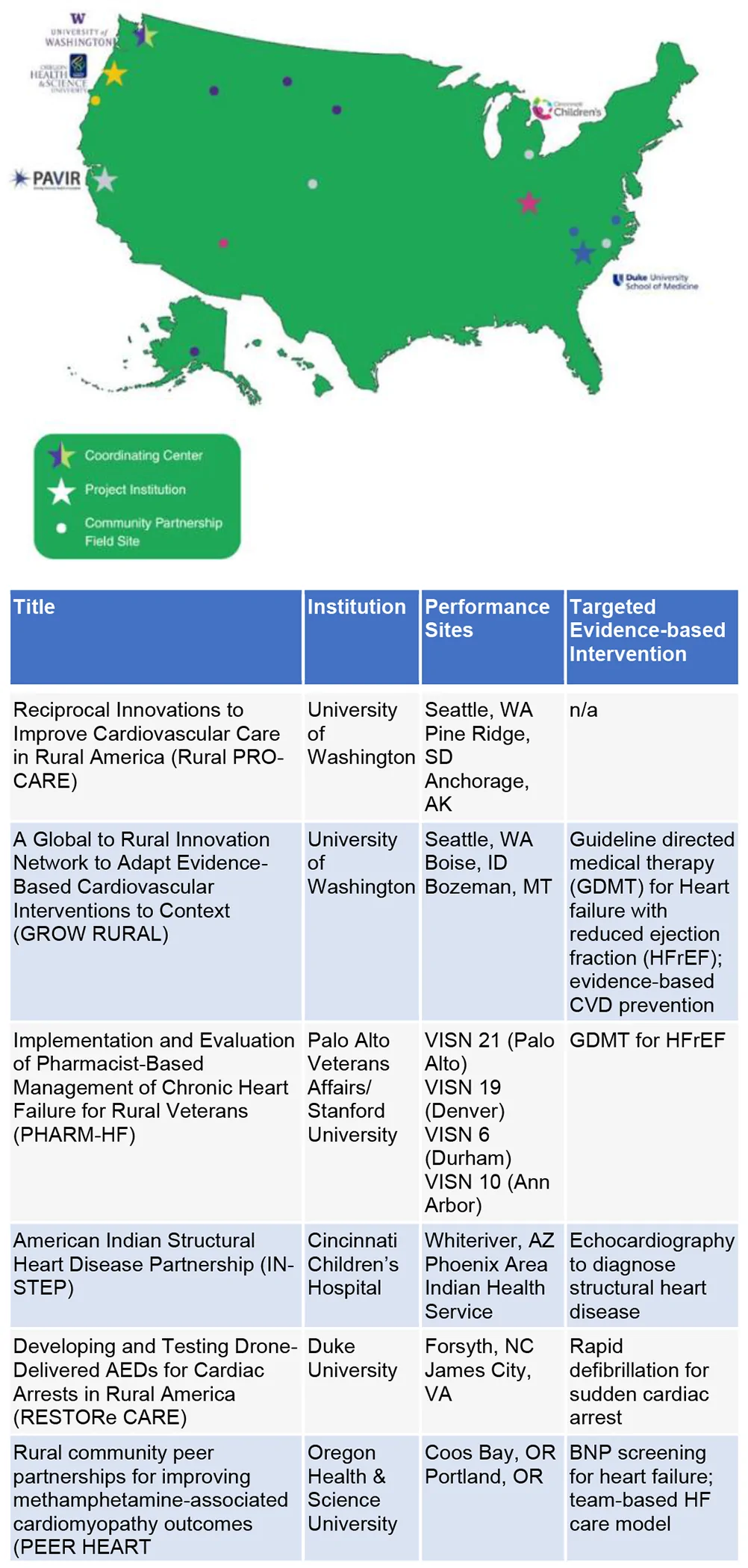
Rural PRO-CARE network

**Figure 3:**
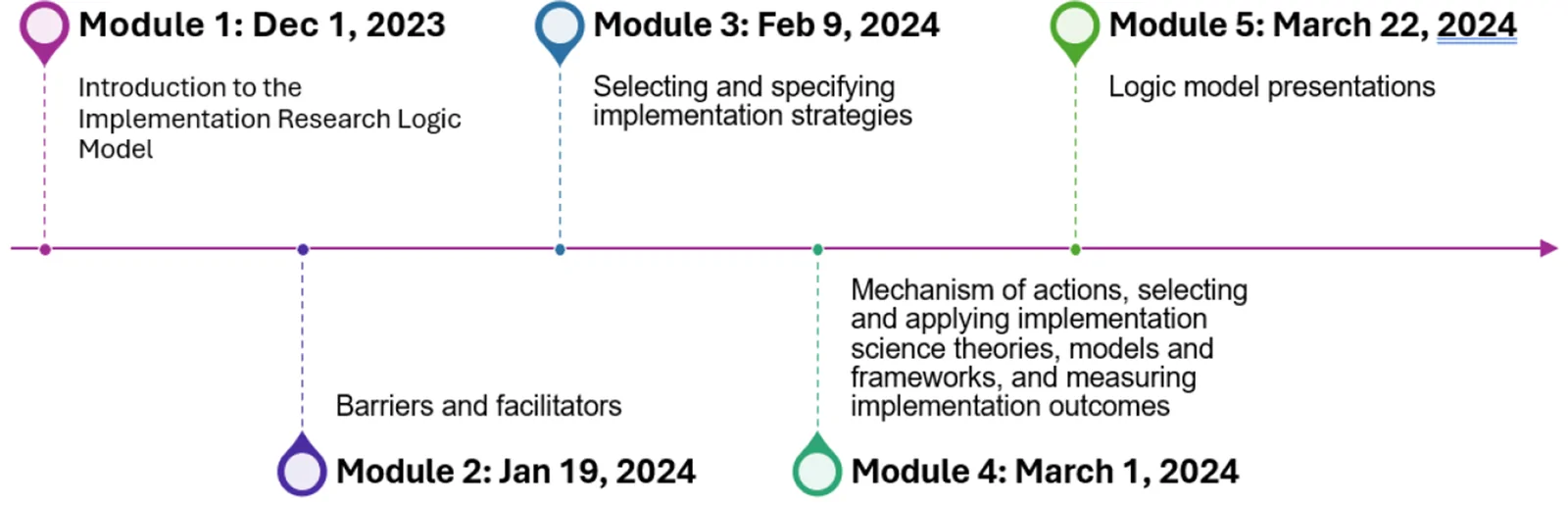
Timeline for IRLM development

**Figure 4:**
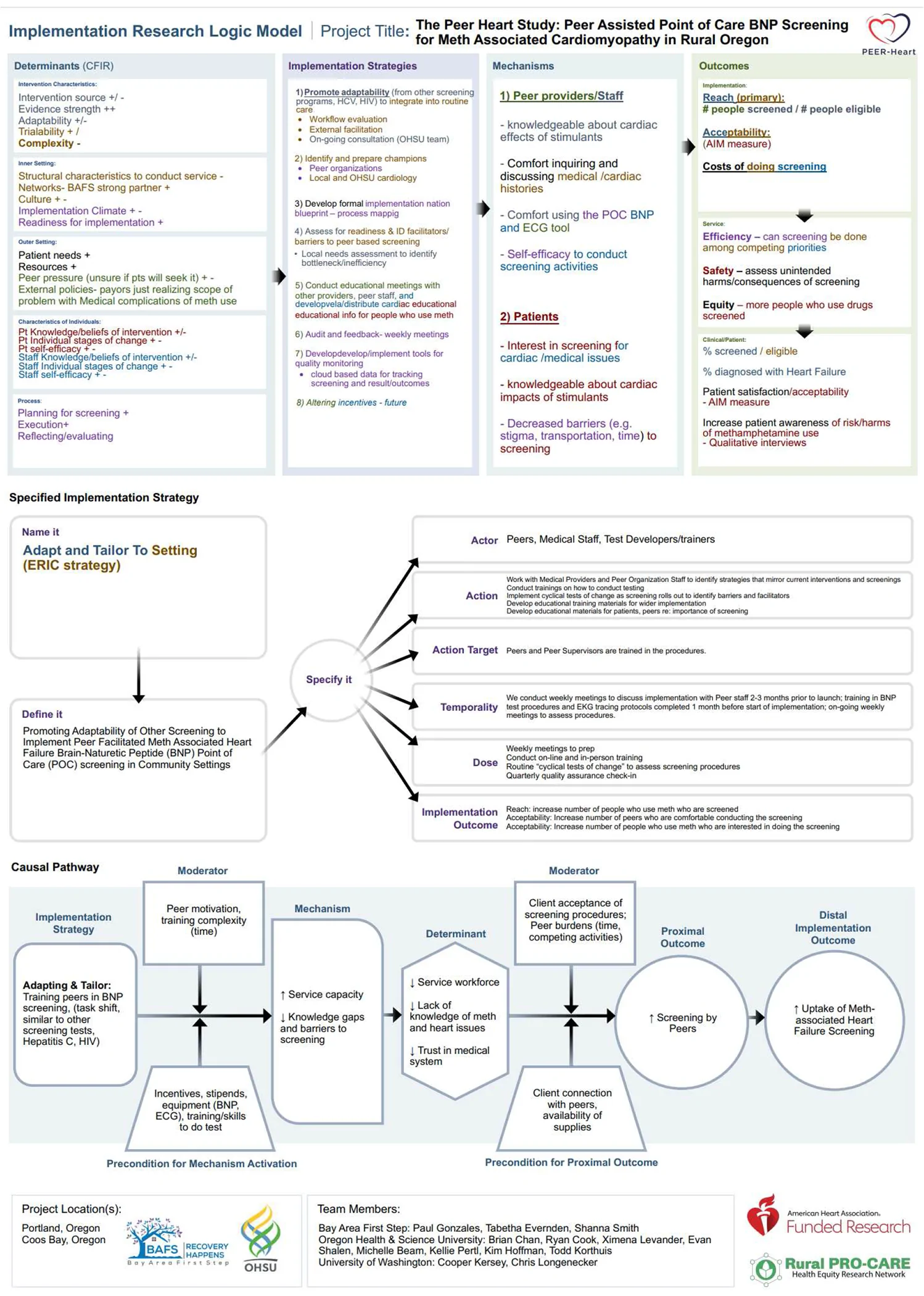
Implementation Research Logic Model for the PEER HEART project

**Table 1:** AAR interview guide

Theme	Question
**1. What occurred?**	During the implementation science skills building and IRLM development process, what actually happened? What were the differences?
**2. What worked and what drove similarities/differences across sites?**	During the implementation science skills building and IRLM development process what worked, what didn’t work and why?
**3. What can be improved next time?**	What will you do differently in the future? (actionable recommendations)

**Table 2:** Planned Use of IRLM

	Theme	How teams planned to use the IRLM	Illustrative quote(s)
1	Use the IRLM as a dynamic tool that allows updating while broader strategies are refined	Teams plan to iteratively return to their IRLM as implementation strategies became clearer, using it as a touchpoint to update barriers, facilitators, and other details.	“And so that’s where we may, over the next few months, learn even some more granular detail about those barriers and facilitators. And so, then we could add those to the IRLM.” — Implementation science expert, RESTORe CARE
2	Use the IRLM to deepen team’s understanding of the drivers of implementation through qualitative inquiry	Teams planned to conduct interviews and other qualitative methods to refine their IRLM and better understand implementation barriers in context.	“We are right now scheduling interviews with some of the pharmacists so that we can dig in deeper…” — Implementation science expert, PHARM-HF
3	Use the IRLM to support teams to test assumptions about mechanisms of action	Teams planned to use the IRLM to specify how strategies were expected to work and to identify what data would be needed to confirm those mechanisms.	“So, we could create a very nice IRLM that that gets at proposed mechanisms of how these strategies are going to work…” — Principal investigator, GROW RURAL
4	IRLM provides a structure to plan implementation measurement, data collection, and analysis planning	Teams planned to use the IRLM to decide what determinants, moderators, and implementation outcomes should be measured during the study.	“I think that the IRLM helps us specifically say, what do we need to measure in terms of implementation and determinants and moderators and that sort of thing…” — Principal investigator, GROW RURAL “It is helping as we think about the project implementation, the theories and frameworks that we can apply.” — Principal Investigator, PEER HEART
7	Use the IRLM to assess readiness before rollout	IRLM encouraged projects to conduct needs assessments and readiness interviews before implementation.	“Yeah. So what we are working on and close to completing is this needs assessment and interviews following that…” — Principal Investigator, RESTORe CARE
8	Use the IRLM to keep implementation efforts focused and on track	Teams planned to use the IRLM as a guide for staying aligned with project goals and for adjusting the emphasis of strategies over time.	“it still feels very applicable. It’s not like we have completely diverged from this…. looking at mechanisms and looking at implementation strategies.” — Fellow, INSTEP
9	IRLM helped teams prioritize strategies	Teams planned to use the IRLM to identify which implementation strategies were receiving enough attention and which needed to be strengthened.	“…we have definitely started to apply more weight to some than others… we haven’t done as much raising community awareness, and we’re kind of starting to re-center on that.” — Principal Investigator, INSTEP
10	IRLM promotes sites to think beyond “one size fits all”	Teams used the IRLM to recognize that implementation should not look identical across settings and that rollout needed to be adapted between sites.	“…executing this the same way that we had in ‘City X’ may not be the correct approach, but there may be things that we want to additionally tailor or think about…” — Principal investigator, RESTORe CARE

## Data Availability

All data generated or analyzed during this study are included in this published article and its supplementary information files.

## List of abbreviations

AHA: American Heart Association
CFIR: Consolidated Framework for Implementation Research
ERIC: Expert Recommendations for Implementing Change
HERN: Health Equity Research Network
GROW RURAL: A Global to Rural Innovation Network to Adapt Evidence Based Cardiovascular Interventions to Context
INSTEP: Implementation and Scale-up of the American Indian Structural Heart Disease Partnership
IRLM: Implementation Research Logic Model
NHLBI: National Heart, Lung, and Blood Institute
NCI: National Cancer Institute
PEER HEART: Rural Community Peer Partnerships for Improving Methamphetamine-Associated Heart Failure Screening and Engagement
PHARM HF: Implementation and Evaluation of Pharmacist-Based Management of Chronic Heart Failure for Rural Veterans
RESTORE CARE: Developing and Testing Drone-Delivered AEDs for Cardiac Arrests in America
RURAL PRO-CARE: ReciPROcal Innovations to Improve Cardiovascular Care in Rural America
SFRN: Strategically Focused Research Network
