# Supplementary material for: Applying the Implementation Research Logic Model (IRLM) as a research planning tool in a multi-project, rural health initiative to improve cardiovascular health service delivery outcomes and quality
Source: Res Sq. 2026 Oct 6:rs.3.rs-10584584. Preprint. [Version 1] doi: 10.21203/rs.3.rs-10584584/v1 (PMC13692599; doi:10.21203/rs.3.rs-10584584/v1)
Supplement: Supplementary file 1 [file NIHPPrs10584584v1-supplement-1.pdf]

## Additional files

1. Implementation Research Logic Model Grow Rural project

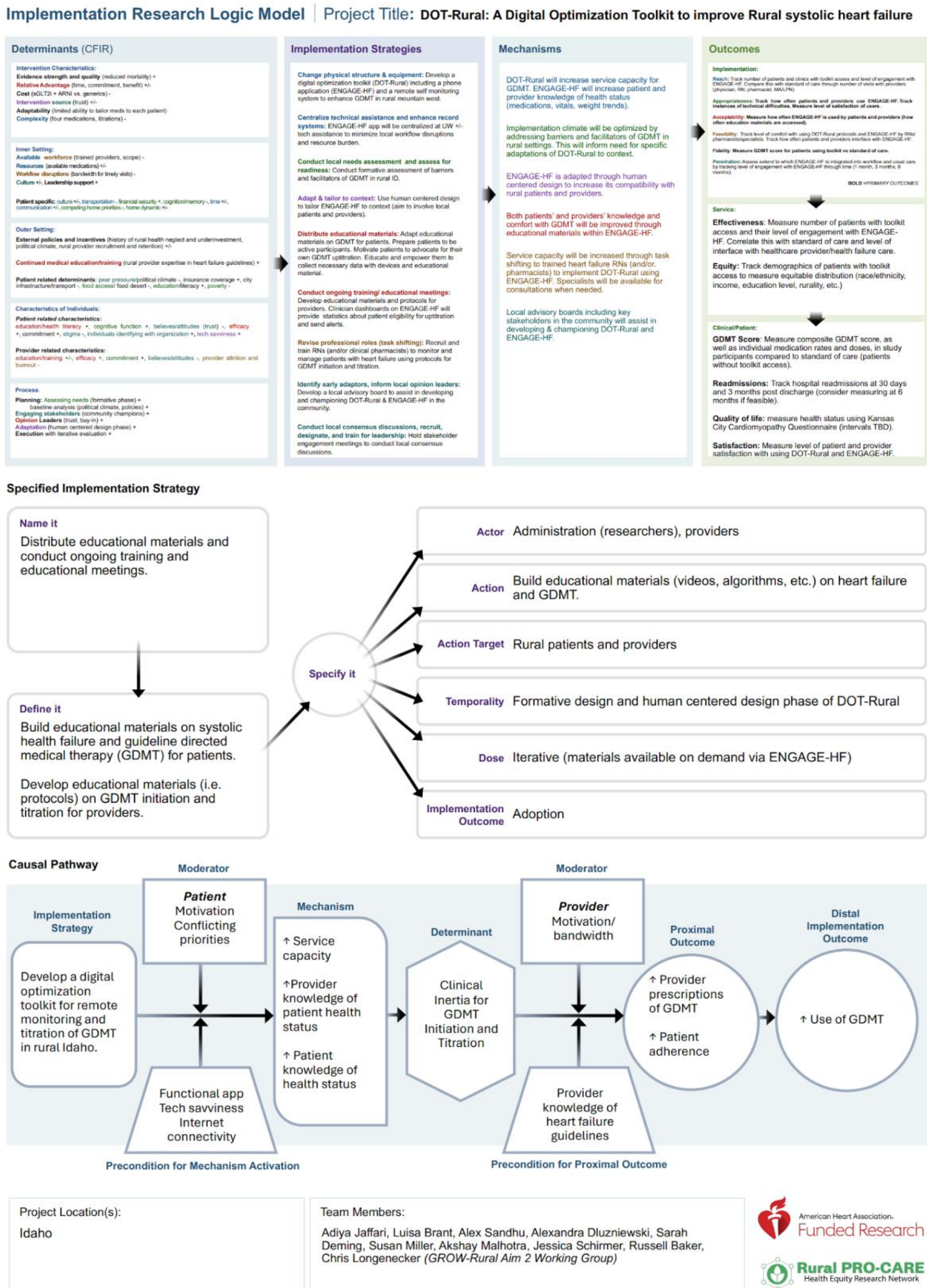


2. Implementation Research Logic Model PHARM HF project

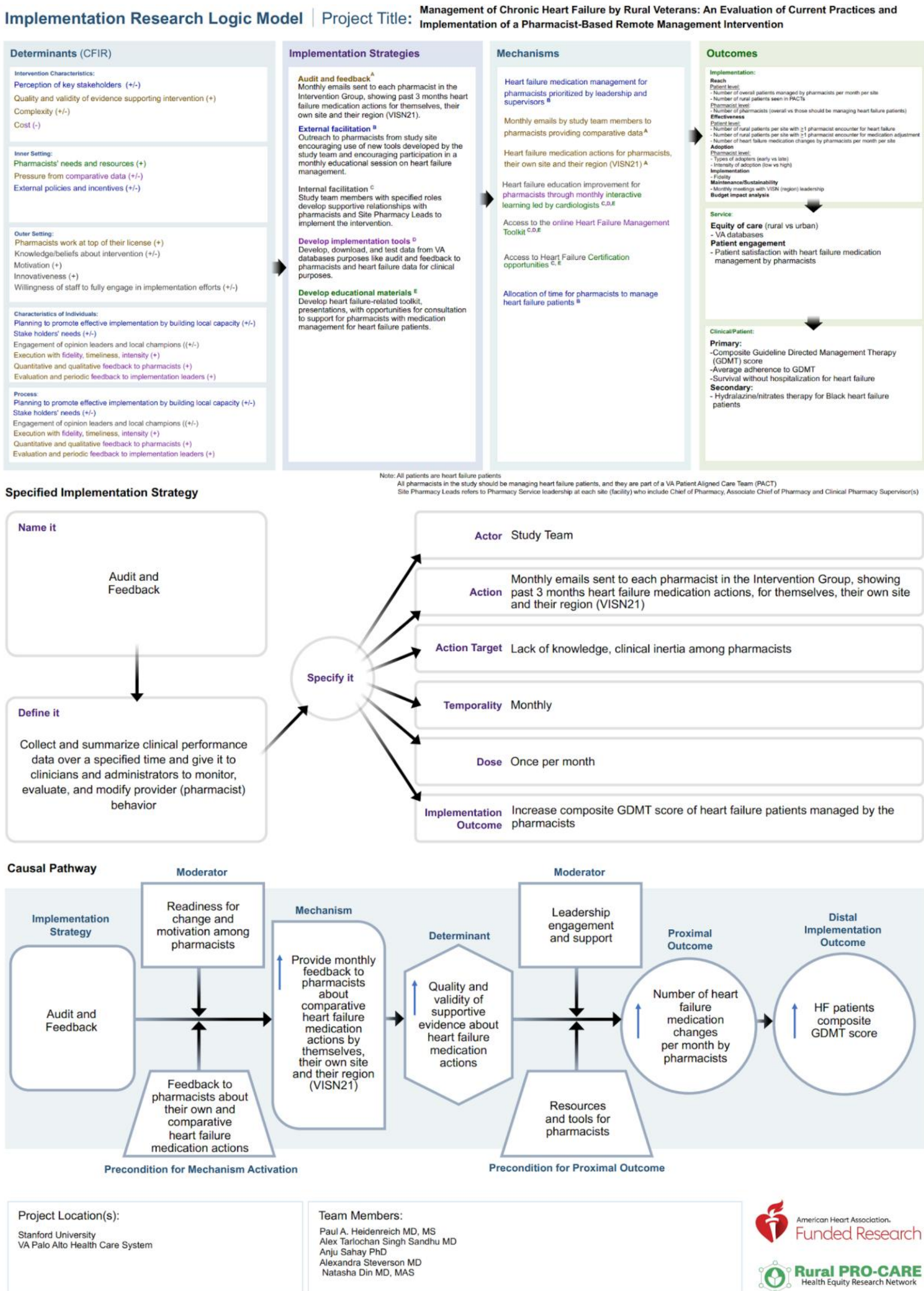

Implementation Research Logic Model
Project Title: Management of Chronic Heart Failure by Rural Veterans: An Evaluation of Current Practices and Implementation of a Pharmacist-Based Remote Management Intervention
Determinants (CFIR)
Intervention Characteristics:
Perception of key stakeholders (+/-)
Quality and validity of evidence supporting intervention (+)
Complexity (+/-)
Cost (-)
Inner Setting:
Pharmacists' needs and resources (+)
Pressure from comparative data (+/-)
External policies and incentives (+/-)
Outer Setting:
Pharmacists work at top of their license (+)
Knowledge/beliefs about intervention (+/-)
Motivation (+)
Innovativeness (+)
Willingness of staff to fully engage in implementation efforts (+/-)
Characteristics of Individuals:
Planning to promote effective implementation by building local capacity (+/-)
Stake holders' needs (+/-)
Engagement of opinion leaders and local champions ((+/-)
Execution with fidelity, timeliness, intensity (+)
Quantitative and qualitative feedback to pharmacists (+)
Evaluation and periodic feedback to implementation leaders (+)
Process:
Planning to promote effective implementation by building local capacity (+/-)
Stake holders' needs (+/-)
Engagement of opinion leaders and local champions ((+/-)
Execution with fidelity, timeliness, intensity (+)
Quantitative and qualitative feedback to pharmacists (+)
Evaluation and periodic feedback to implementation leaders (+)
Implementation Strategies
Audit and feedback A
Monthly emails sent to each pharmacist in the Intervention Group, showing past 3 months heart failure medication actions for themselves, their own site and their region (VISN21).
External facilitation B
Outreach to pharmacists from study site encouraging use of new tools developed by the study team and encouraging participation in a monthly educational session on heart failure management.
Internal facilitation C
Study team members with specified roles develop supportive relationships with pharmacists and Site Pharmacy Leads to implement the intervention.
Develop implementation tools D
Develop, download, and test data from VA databases purposes like audit and feedback to pharmacists and heart failure data for clinical purposes.
Develop educational materials E
Develop heart failure-related toolkit, presentations, with opportunities for consultation to support for pharmacists with medication management for heart failure patients.
Mechanisms
Heart failure medication management for pharmacists prioritized by leadership and supervisors B
Monthly emails by study team members to pharmacists providing comparative data A
Heart failure medication actions for pharmacists, their own site and their region (VISN21) A
Heart failure education improvement for pharmacists through monthly interactive learning led by cardiologists C,D,E
Access to the online Heart Failure Management Toolkit C,D,E
Access to Heart Failure Certification opportunities C, E
Allocation of time for pharmacists to manage heart failure patients B
Outcomes
Implementation:
Reach
Patient level:
- Number of overall patients managed by pharmacists per month per site
- Number of rural patients seen in PACTs
Pharmacist level:
- Number of pharmacists (overall vs those should be managing heart failure patients)
Effectiveness
Patient level:
- Number of rural patients per site with ≥1 pharmacist encounter for heart failure
- Number of rural patients per site with ≥1 pharmacist encounter for medication adjustment
- Number of heart failure medication changes by pharmacists per month per site
Adoption
Pharmacist level:
- Types of adopters (early vs late)
- Intensity of adoption (low vs high)
Implementation
- Fidelity
Maintenance/Sustainability
- Monthly meetings with VISN (region) leadership
Budget impact analysis
Service:
Equity of care (rural vs urban)
- VA databases
Patient engagement
- Patient satisfaction with heart failure medication management by pharmacists
Clinical/Patient:
Primary:
-Composite Guideline Directed Management Therapy (GDMT) score
-Average adherence to GDMT
-Survival without hospitalization for heart failure
Secondary:
- Hydralazine/nitrates therapy for Black heart failure patients
Note: All patients are heart failure patients
All pharmacists in the study should be managing heart failure patients, and they are part of a VA Patient Aligned Care Team (PACT)
Site Pharmacy Leads refers to Pharmacy Service leadership at each site (facility) who include Chief of Pharmacy, Associate Chief of Pharmacy and Clinical Pharmacy Supervisor(s)
Specified Implementation Strategy
Name it
Audit and Feedback
Define it
Collect and summarize clinical performance data over a specified time and give it to clinicians and administrators to monitor, evaluate, and modify provider (pharmacist) behavior
Specify it
Actor Study Team
Action Monthly emails sent to each pharmacist in the Intervention Group, showing past 3 months heart failure medication actions, for themselves, their own site and their region (VISN21)
Action Target Lack of knowledge, clinical inertia among pharmacists
Temporality Monthly
Dose Once per month
Implementation Outcome Increase composite GDMT score of heart failure patients managed by the pharmacists
Causal Pathway
Implementation Strategy
Audit and Feedback
Moderator
Readiness for change and motivation among pharmacists
Feedback to pharmacists about their own and comparative heart failure medication actions
Precondition for Mechanism Activation
Mechanism
Provide monthly feedback to pharmacists about comparative heart failure medication actions by themselves, their own site and their region (VISN21)
Determinant
Quality and validity of supportive evidence about heart failure medication actions
Moderator
Leadership engagement and support
Resources and tools for pharmacists
Precondition for Proximal Outcome
Proximal Outcome
Number of heart failure medication changes per month by pharmacists
Distal Implementation Outcome
HF patients composite GDMT score
Project Location(s):
Stanford University
VA Palo Alto Health Care System
Team Members:
Paul A. Heidenreich MD, MS
Alex Tarlochan Singh Sandhu MD
Anju Sahay PhD
Alexandra Steverson MD
Natasha Din MD, MAS
American Heart Association. Funded Research
Rural PRO-CARE Health Equity Research Network

3. Implementation Research Logic Model INSTEP project

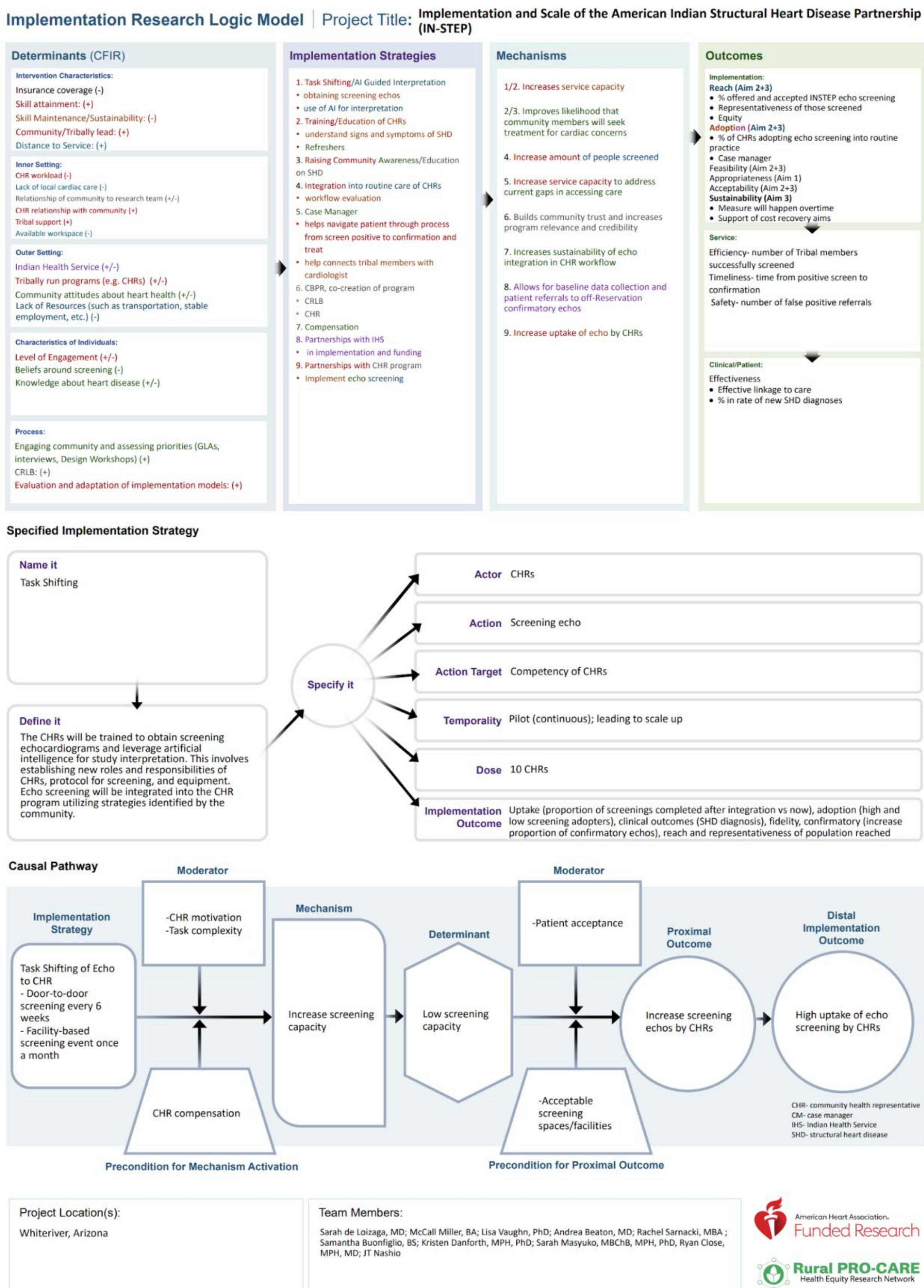
Implementation Research Logic Model
Project Title: Implementation and Scale of the American Indian Structural Heart Disease Partnership (IN-STEP)
Determinants (CFIR)
Intervention Characteristics:
Insurance coverage (-)
Skill attainment: (+)
Skill Maintenance/Sustainability: (-)
Community/Tribally lead: (+)
Distance to Service: (+)
Inner Setting:
CHR workload (-)
Lack of local cardiac care (-)
Relationship of community to research team (+/-)
CHR relationship with community (+)
Tribal support (+)
Available workspace (-)
Outer Setting:
Indian Health Service (+/-)
Tribally run programs (e.g. CHRs) (+/-)
Community attitudes about heart health (+/-)
Lack of Resources (such as transportation, stable employment, etc.) (-)
Characteristics of Individuals:
Level of Engagement (+/-)
Beliefs around screening (-)
Knowledge about heart disease (+/-)
Process:
Engaging community and assessing priorities (GLAs, interviews, Design Workshops) (+)
CRLB: (+)
Evaluation and adaptation of implementation models: (+)
Implementation Strategies
1. Task Shifting/AI Guided Interpretation
• obtaining screening echos
• use of AI for interpretation
2. Training/Education of CHRs
• understand signs and symptoms of SHD
• Refreshers
3. Raising Community Awareness/Education on SHD
4. Integration into routine care of CHRs
• workflow evaluation
5. Case Manager
• helps navigate patient through process from screen positive to confirmation and treat
• help connects tribal members with cardiologist
6. CBPR, co-creation of program
• CRLB
• CHR
7. Compensation
8. Partnerships with IHS
• in implementation and funding
9. Partnerships with CHR program
• Implement echo screening
Mechanisms
1/2. Increases service capacity
2/3. Improves likelihood that community members will seek treatment for cardiac concerns
4. Increase amount of people screened
5. Increase service capacity to address current gaps in accessing care
6. Builds community trust and increases program relevance and credibility
7. Increases sustainability of echo integration in CHR workflow
8. Allows for baseline data collection and patient referrals to off-Reservation confirmatory echos
9. Increase uptake of echo by CHRs
Outcomes
Implementation:
Reach (Aim 2+3)
• % offered and accepted INSTEP echo screening
• Representativeness of those screened
• Equity
Adoption (Aim 2+3)
• % of CHRs adopting echo screening into routine practice
• Case manager
Feasibility (Aim 2+3)
Appropriateness (Aim 1)
Acceptability (Aim 2+3)
Sustainability (Aim 3)
• Measure will happen overtime
• Support of cost recovery aims
Service:
Efficiency- number of Tribal members successfully screened
Timeliness- time from positive screen to confirmation
Safety- number of false positive referrals
Clinical/Patient:
Effectiveness
• Effective linkage to care
• % in rate of new SHD diagnoses
Specified Implementation Strategy
Name it
Task Shifting
Define it
The CHRs will be trained to obtain screening echocardiograms and leverage artificial intelligence for study interpretation. This involves establishing new roles and responsibilities of CHRs, protocol for screening, and equipment. Echo screening will be integrated into the CHR program utilizing strategies identified by the community.
Specify it
Actor CHRs
Action Screening echo
Action Target Competency of CHRs
Temporality Pilot (continuous); leading to scale up
Dose 10 CHRs
Implementation Outcome Uptake (proportion of screenings completed after integration vs now), adoption (high and low screening adopters), clinical outcomes (SHD diagnosis), fidelity, confirmatory (increase proportion of confirmatory echos), reach and representativeness of population reached
Causal Pathway
Implementation Strategy
Task Shifting of Echo to CHR
- Door-to-door screening every 6 weeks
- Facility-based screening event once a month
Moderator
-CHR motivation
-Task complexity
CHR compensation
Precondition for Mechanism Activation
Mechanism
Increase screening capacity
Determinant
Low screening capacity
Moderator
-Patient acceptance
-Acceptable screening spaces/facilities
Precondition for Proximal Outcome
Proximal Outcome
Increase screening echos by CHRs
Distal Implementation Outcome
High uptake of echo screening by CHRs
CHR- community health representative
CM- case manager
IHS- Indian Health Service
SHD- structural heart disease
Project Location(s):
Whiteriver, Arizona
Team Members:
Sarah de Loizaga, MD; McCall Miller, BA; Lisa Vaughn, PhD; Andrea Beaton, MD; Rachel Sarnacki, MBA ; Samantha Buonfiglio, BS; Kristen Danforth, MPH, PhD; Sarah Masyuko, MBChB, MPH, PhD, Ryan Close, MPH, MD; JT Nashio
American Heart Association. Funded Research
Rural PRO-CARE Health Equity Research Network

## 4. Implementation Research Logic Model RESTORE CARE project

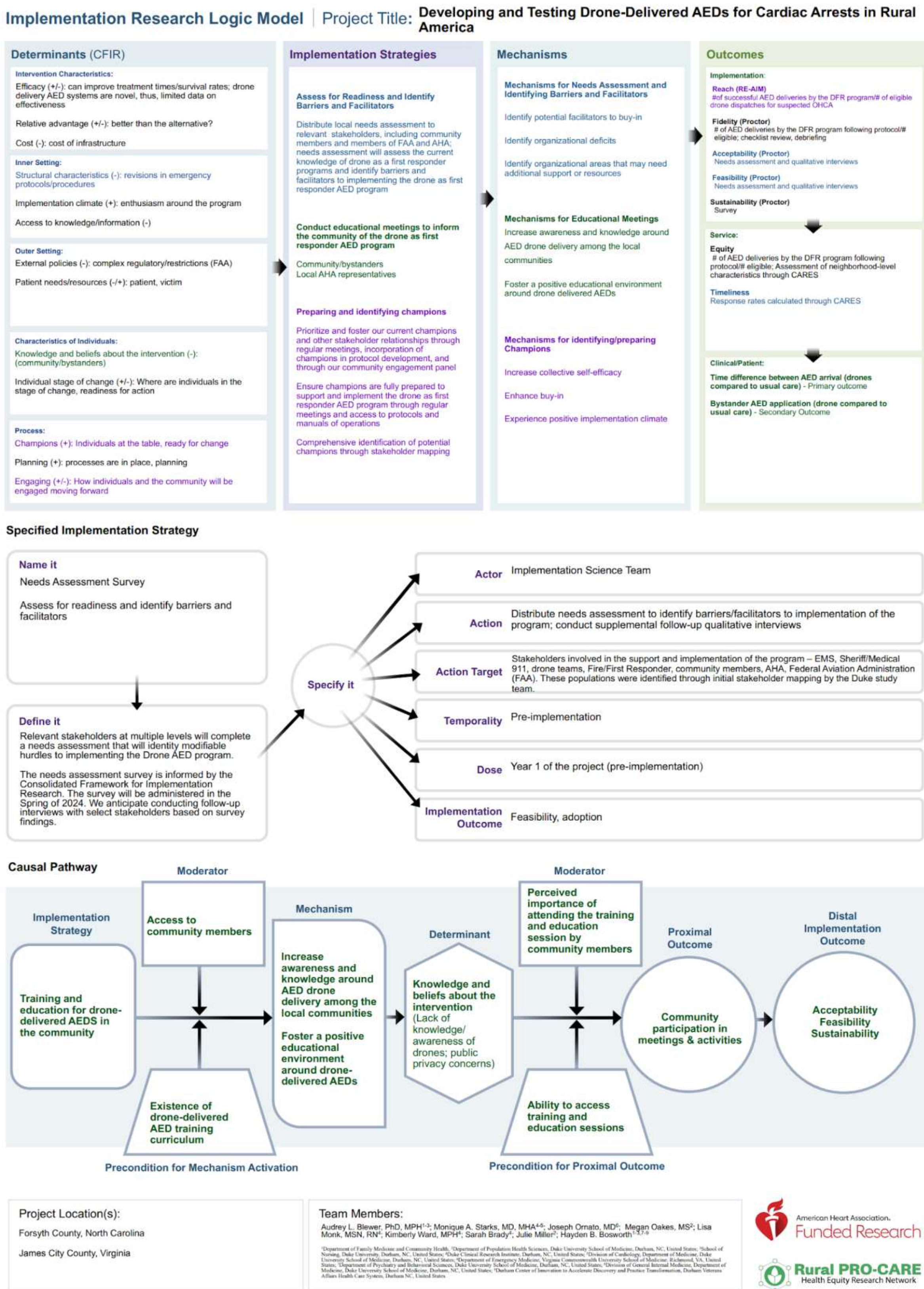

**5. Consensus standards for the reporting of organisational case studies**

| Reporting item | Page number on which item was reported | Page number of justification for not reporting |
|---|---|---|
| **Describing the design** | | |
| 1. Define the research as a case study | 4 | |
| 2. State the broad aims of the study | 4 | |
| 3. State the research question(s)/hypotheses | 4 | |
| 4. Identify the specific case(s) and justify the selection | 5-6 | |
| **Describing the data collection** | | |
| 5. Describe how data were collected | 8-10 | |
| 6. Describe the sources of evidence used | 8-10 | |
| 7. Describe any ethical considerations and obtainment of relevant approvals, access and permissions | 26 | |
| **Describing the data analysis** | | |
| 8. Describe the analysis methods | 10-11 | |
| **Interpreting the results** | | |

| Reporting item | Page number on which item was reported | Page number of justification for not reporting |
| --- | --- | --- |
| 9. Describe any inherent shortcomings in the design and analysis and how these might have influenced the findings | 24-25 | |
| 10. Consider the appropriateness of methods used for the question and subject matter and why it was that qualitative methods were appropriate | 11 | |
| 11. Discuss the data analysis | 11-24 | |
| 12. Ensure that the assertions are sound, neither over- nor under-interpreting the data | 22-24 | |
| 13. State any caveats about the study | 23-24 | |
